# ARAP1 negatively regulates stress fibers formation and metastasis in lung adenocarcinoma via controlling Rho signaling

**DOI:** 10.1007/s12672-023-00832-x

**Published:** 2023-11-27

**Authors:** Zhengzheng Zhang, Wenran Xie, Bojiang Gong, Xue Liang, Hongjia Yu, Yanwen Yu, Zhixiong Dong, Fanggui Shao

**Affiliations:** 1 Department of Laboratory Medicine, the First Affiliated Hospital of Wenzhou Medical University, Wenzhou, 325000 Zhejiang People’s Republic of China; 2 Zhejiang Provincial Key Laboratory of Medical Genetics, Key Laboratory of Laboratory Medicine, Ministry of Education, School of Laboratory Medicine and Life Science, Wenzhou Medical University, University Town, Chashan, 325000 Wenzhou, Zhejiang People’s Republic of China; 3 Department of Intensive Care Unit, the First Affiliated Hospital of Wenzhou Medical University, 2 Fuxue Lane, Wenzhou, 325000 Zhejiang China; 4 Department of Clinical Laboratory, Key Laboratory of Clinical Laboratory Diagnosis and Translational Research of Zhejiang Province, the First Affiliated Hospital of Wenzhou Medical University, Wenzhou, 325000 Zhejiang People’s Republic of China

**Keywords:** ARAP1, GTPase, Lung adenocarcinoma, Metastasis, Rho, Stress fiber

## Abstract

Small GTPases regulate multiple important cellular behaviors and their activities are strictly controlled by a mass of regulators. The dysfunction or abnormal expression of small GTPases or their regulators was frequently observed in various cancers. Here, we analyzed the expression and prognostic correlation of several GTPases and related regulators based on the TCGA database and found that Ankyrin Repeat and PH Domain 1 (ARAP1), a GTPase activating protein (GAP), is reduced in lung adenocarcinoma tissues compared to normal tissues and displays a positive correlation with overall survival (OS) and progression-free survival (PFS) of patients with lung adenocarcinoma. qPCR and western blot verified that ARAP1 is frequently downregulated in lung adenocarcinoma tumor tissues and cancer cells, and its downregulation might be mediated by epigenetic modification. Moreover, metastatic assays showed that overexpression of ARAP1 significantly inhibits metastasis of lung adenocarcinoma in vitro and in vivo. We further demonstrated that Rho signaling inhibition, mediated by RhoGAP activity of ARAP1, majorly contributes to suppressing migration and invasion of lung adenocarcinoma cancer cells via inhibiting stress fibers formation. In summary, this study indicates that ARAP1 may serve as a potential prognostic predictor and a metastatic suppressor in lung adenocarcinoma via its RhoGAP activity.

**Supplementary Information:**

The online version contains supplementary material available at 10.1007/s12672-023-00832-x.

## Introduction

Lung cancer remains one of the most common cancers and the leading cause of cancer mortality worldwide [1], which is comprised of non-small cell lung cancer (NSCLC) and small-cell lung cancer (SCLC). NSCLC accounts for about 85% of all diagnosed lung cancers and is further sorted into three main subtypes: large cell carcinoma, squamous cell carcinoma, and adenocarcinoma [2]. Among them, lung adenocarcinoma (LUAD) is the most common subtype of NSCLC and accounts for about 40% of lung cancers. Despite obvious advances in diagnosis and therapy, the 5 years survival of NSCLC patients remains not satisfied, particularly for patients with advanced NSCLC was estimated to be only 6% [3]. Thus, it is vital to confirm the disease via earlier diagnosis and develop a reasonable therapeutic regimen for improving the survival rate.

Metastasis is the leading cause of mortality in patients with solid tumors [4]. NSCLC has been reported to frequently metastasize to the contralateral lung, bone, or brain, leading to worse survival [5]. Therefore, it is significant to develop effective therapeutic schedules for impeding NSCLC metastasis. Cell metastasis involves diverse cellular processes, including local invasion and intravasation into the blood system, survival and migration in circulation, and extravasation to seed a new tumor in metastatic sites [6]. In the past two decades, a large number of studies have demonstrated that epithelial-mesenchymal transition (EMT) plays a critical role in cancer metastasis [7]. EMT is usually characterized by decreased epithelial marker E-cadherin and enhanced expression of mesenchymal markers N-cadherin, Vimentin and MMP9 [8]. In addition, cancer cells undergoing EMT require rearrangements of actin filaments, such as the formation of stress fibers and pseudopodia [9–11].

Small GTPases, a kind of key molecular switches, regulate numerous cell functions via switching between GDP-bound inactive and GTP-bound active forms. The switch is mediated by various regulators including Guanine nucleotide exchange factors (GEFs), GTPase activating proteins (GAPs) and guanine nucleotide dissociation inhibitors (GDIs) [12, 13]. Ras homology (Rho) GTPase is one of the most crucial small GTPase families involved in a wide range of tumor-related processes, such as malignant transformation, cell cycle progression, cell polarity, cytoskeleton dynamics, as well as cell migration and invasion [14, 15]. Plenty of studies have discovered altered expression or mutation of Rho GTPases and related regulators in malignant [16], which serve as a viable therapeutic strategy in cancer treatment. Upon activation, Rho can interact with downstream effectors to transmit signaling. For instance, Rho Kinase (ROCK), a serine/threonine kinase downstream of Rho, promotes the formation of RhoA-induced stress fibers and focal adhesions through phosphorylation of downstream targets, including LIM kinase, myosin light chain (MLC) and Myosin Phosphatase-Targeting Subunit 1 (MYPT1) [17–19]. It has been widely accepted that Rho GTPases play a vital role in forming actin-rich structures such as protrusions, stress fiber and pseudopodia, which provide the essential force for cell motility [20].

We sought to screen abnormality in small GTPases and their regulators in LUAD. We found that ArfGAP with RhoGAP Domain, Ankyrin Repeat and PH Domain 1 (*ARAP1*) mRNA is frequently reduced in tumor tissues, and its lower expression correlates with worse prognosis of patients with LUAD. ARAP1 is a member of ARAP family with both Arf GAP and Rho GAP activities, by which ARAP1 makes a direct link between ADP-ribosylation factor (Arf) GTPases and Rho GTPases signaling [21]. Although it has been reported that ARAP1 participates in regulating epidermal growth factor receptor (EGFR) endocytosis and ARAP1 is significantly lower in high-grade serous carcinoma (HGSC) tumor tissues of patients with early progression compared to those with late [22–25], the function of ARAP1 in tumorigenesis remains elusive and needs to further investigate. Here, we explored the role of ARAP1 in LUAD tumorigenesis and demonstrated that ARAP1 inhibits cell metastasis mainly by suppressing Rho GTPase-mediated stress fibers formation in LUAD.

## Materials and methods

### Human tissues collection

We collected paired tissues from tumor and adjacent non-cancerous tissues of thirty-eight LUAD patients were supplied by The First Affiliated Hospital of Wenzhou Medical University (China). Written informed consent was obtained from the participants. The samples were used to extract total RNA and protein.

### Plasmid construction and viral infection

Human ARAP1 cDNA was cloned into a pCDH-CMV-3 × Flag vector (a gift from Yu Zhang, Northeast Normal University) or pEGFP-N1 vector (Addgene) by using the ClonExpress II Kit (Vazyme). Mutagenesis was performed using inverse PCR.

All of the lentivirus vectors were packaged in 293 T cells with the psPAX2 and pMD2.G packaging vectors. Lentiviruses were used to infect A549 and HCC827 cells. Stably transfected cell lines were selected by puromycin for 2 days.

### Cell culture and transfection

Lung adenocarcinoma cells A549 and HCC827 were generous gifts from Dr. Haishang Huang of Wenzhou Medical University (Wenzhou, China). A549 cells were cultured in Ham's F-12 K (Gibco) medium containing 10% fetal bovine serum (FBS, Gibco), and HCC827 cells were grown in DMEM medium (Gibco) supplemented with 10% FBS. All cells were cultured at 37 °C with 5% CO_2_. Lipofectamine 3000 (Life Technologies) was used for plasmids or siRNAs transfection.

### Western blot and immunofluorescence analysis

Cells were harvested or fixed for western blot or immunofluorescence analysis as previously described [26]. Briefly, cells were lysed and equal amounts of total protein lysates were used for SDS-PAGE. Then performed western blot with indicated antibodies (Table S1). For immunofluorescence assay, cells were cultured on poly-L-lysine-coated coverslips and fixed with formaldehyde, and then immunostained with indicated antibodies (Table S1). DAPI was used for DNA staining. Images were captured using a NIKON A1 confocal microscope (Nikon, Tokyo, Japan). FITC-conjugated phalloidin was obtained from Sigma-Aldrich (P5282, Billerica, USA).

### CCK8 assay and colony formation

For CCK8 assay, cells were planted in 96-well plates (1000 cells per well) and grown at indicated time. Cells proliferation was determined by adding CCK8 solution (Dojindo, Kyushu, Japan) for 2 h and measuring the absorbance at 450 nm. For colony formation analysis, cells were seeded in 6-well plates (300 cells per well) and cultured for 10 days, and then fixed with 4% paraformaldehyde and stained with 0.1% crystal violet [27].

### Wound healing assay, transwell migration and invasion assay

Cell migration and invasion assays were performed as previously described [28]. For wound healing assay, ARAP1 and its mutants stably overexpressed cells or control cells (1 × 10^6^ cells per well) were planted on 6-well plates and cultured for 24 h. Then the cells were cultured overnight in serum-free medium for starvation. A 200 µl pipette tip was used to make a wound. Wound closure caused by cell migrating was photographed. The area of the wound was analyzed by Image J software (NIH, USA). Wound closure rate was calculated as a percentage of initial sizes. For transwell migration and invasion assays, 1 × 10^5^ starved cells were suspended in serum-free medium and were seeded to the upper chambers of transwell (Corning, NY, USA) with or without matrigel, and media containing 20% FBS were added to the bottom chambers. After 24 h incubation, the migrated or invaded cells were fixed in 4% paraformaldehyde and stained with 0.1% crystal violet. The number of migrating cells in 5 random fields was counted under 10 × magnification by Image J software, and the means for all fields in a chamber relative to control group were calculated. The relative changes in wound healing or metastatic ability of Flag-ARAP1 expressed cells to control cells were calculated.

### Tumor metastasis assay in vivo

Animal experiments were approved by the Animal Ethics Committee of Wenzhou Medical University (WMU). We performed animal experiments in the SPF environment in the Animal Center of WMU. 4–5-weeks-old male BALB/c nude mice were purchased from Vital River Laboratories (Zhejiang, China). 2 × 10^6^ cells were injected into the tail vein of each mouse. After 7 weeks, all mice were sacrificed by euthanasia. The lungs of each BALB/c nude mouse were isolated, and visible nodules on the surface of the lungs were counted. Then, the lungs were formalin-fixed, paraffin-embedded and stained with hematoxylin and eosin (H&E) [29]. The area of tumor in each H&E image was measured using Image J, and the ratio of tumor area to lung area was calculated.

### RNA extraction and quantitative RT-PCR (qRT-PCR)

RNA extraction and qRT-PCR were performed as previous depicted [30]. Briefly, total RNA from siRNAs or plasmids transfected cells was extracted with Trizol reagent (Invitrogen). One microgram RNA was used for cDNA synthesis and qPCR was performed on a Biorad CFX 96 Touch using SYBR Green (TIANGEN BIOTECH, Beijing, China) as a dsDNA-specific fluorescent dye. *GAPDH* was used for standardizing indicated mRNA level [31]. Primers used for qPCR amplification were as follows [32–34]: *ARAP1*: 5′-GGGACCAGAAGTTTGAAGTGA-3′, 5′-CCACGTACAGCTTATTCTTGAA-3′; *GAPDH*: 5′-TTCATTGACCTCAACTACATGGTTTAC-3′, 5′-TGACAAGCTTCCCGTTCTCA-3′; *RhoA*: 5′-CTGGTGATTGTTGGTGATGG-3′, 5′-GCGATCATAATCTTCCTGCC-3′; *RhoB*: 5′-TGCTGATCGTGTTCAGTAAG-3′, 5′-AGCACATGAGAATGACGTCG-3′; *RhoC*: 5′-TCCTCATCGTCTTCAGCAAG-3′, 5′-GAGGATGACATCAGTGTCCG-3′.

### RhoA and RhoC GTPase activity assays

RhoA and RhoC GTPase activities were determined by using Active GTPase Kits (Cell Signaling Technology) according to the manufacturer’s instructions. Briefly, cells were harvested and lysed in lysis buffer, and the activated RhoA-GTP or RhoC-GTP was bound to the GST-Rhotekin RBD fusion protein, which can then be pulled down by glutathione resin. After precipitation, samples were subjected to immunoblotting with indicated antibodies.

### Statistical analysis

All of the statistical analyses were performed using GraphPad Prism 8.0 and SPSS 20.0 statistical software. Data are presented as the means ± standard deviations from at least 3 independent experiments. *P* < 0.05 is considered statistically significant. Chi-square test was used to analyze the correlation between *ARAP1* expression and clinical features.

## Results

### ARAP1 is frequently reduced in LUAD with unfavorable prognosis of LUAD patients.

**Fig. 1:**
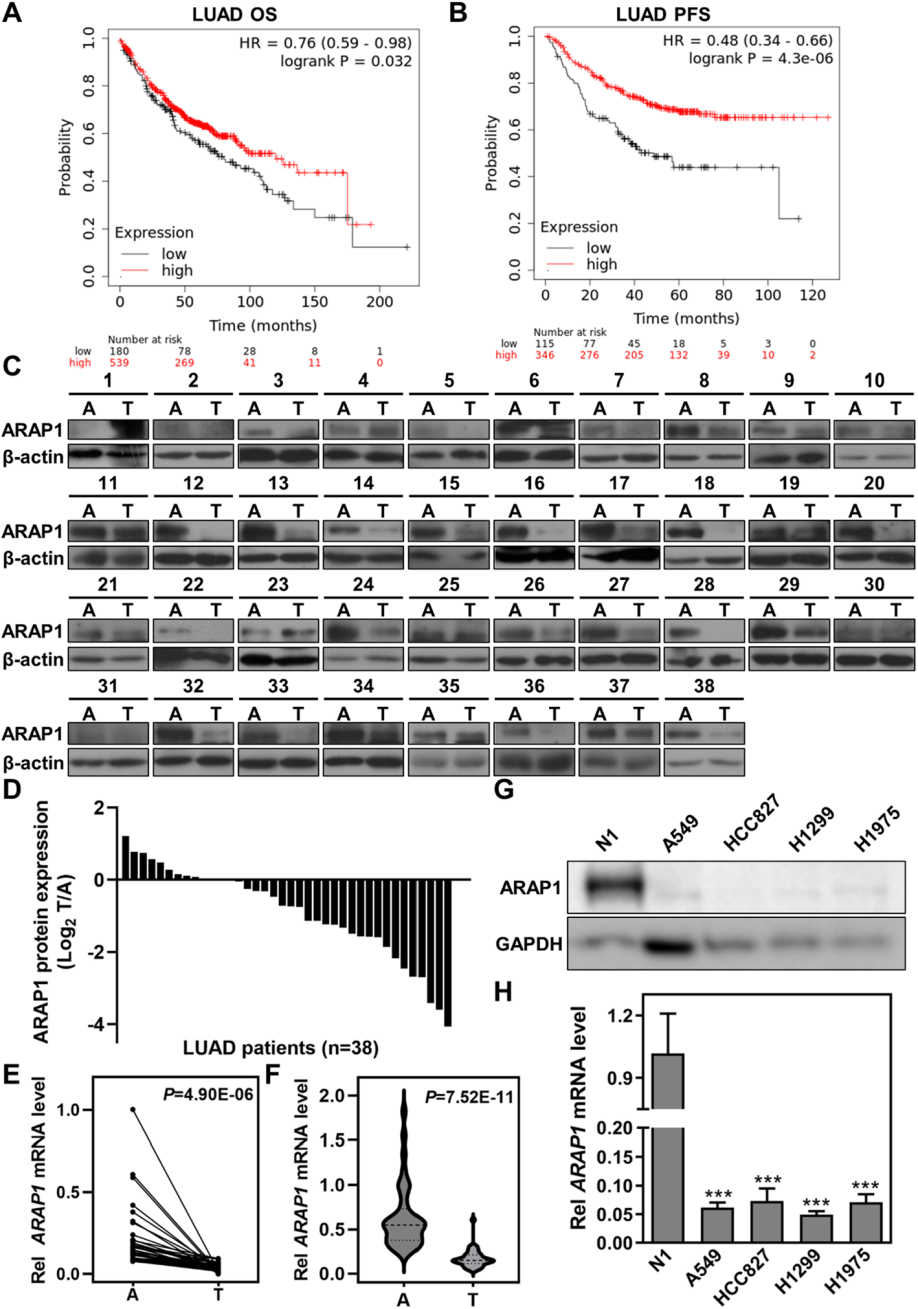
Ankyrin Repeat and PH Domain 1 (ARAP1) is frequently down-regulated with a worse prognosis in lung adenocarcinoma (LUAD) patients. TCGA data analysis showed that ARAP1 expression is negatively correlated with poor OS (**A**) and PFS (**B**) of LUAD patients. Western blot (**C**) or qRT-PCR (**E**, **F**) examined the expression of ARAP1 in tumor and paired adjacent non-cancerous tissues of LUAD patients. T: tumor; A: paired adjacent non-cancerous. (**D**) The density of bands in C was quantified using Image J and relative expression to loading control was calculated. Western blot (**G**) and qRT-PCR (**H**) detected ARAP1 protein and mRNA level in different LUAD cancer cells and a normal lung epithelial tissue. *ARAP1* mRNA level was normalized to the expression of *GAPDH* (mean ± SD, n = 3). ***P < 0.001 compared to N1 by Student *t* test

To explore the critical regulator(s) in LUAD tumorigenesis, we sought to screen and analyze the expression and prognostic correlation of small GTPases and their regulators using the online database (UALCAN: http://ualcan.path.uab.edu/index.html; Kaplan–Meier Plotter: https://kmplot.com/analysis/). We identified that both mRNA and protein expression of ARAP1 were reduced in LUAD tumor tissues in comparison to normal tissues (Fig. S1A, B). Moreover, analysis of Kaplan–Meier survival datasets showed that lower expression of ARAP1, even in tumor tissues of early-stage, was closely associated with poorer overall survival (OS) and progression-free survival (PFS) in LUAD patients (Fig. 1A, B and S1C, D). To investigate the possible role of ARAP1 in LUAD, we examined ARAP1 expression in patients with LUAD (Table S2). The results revealed that both protein and mRNA expression of ARAP1 were frequently decreased in tumor samples as compared with the adjacent normal counterparts (Fig. 1C–F). Accordingly, compared with a case of normal lung tissue, ARAP1 was dramatically down-regulated in a panel of LUAD cell lines (Fig. 1G, H). Together, these results indicate that ARAP1 is reduced in LUAD tumorigenesis and positively correlated with a favorable prognosis of LUAD patients.

### ARAP1 expression in LUAD cells might be regulated by epigenetic modifications

**Fig. 2:**
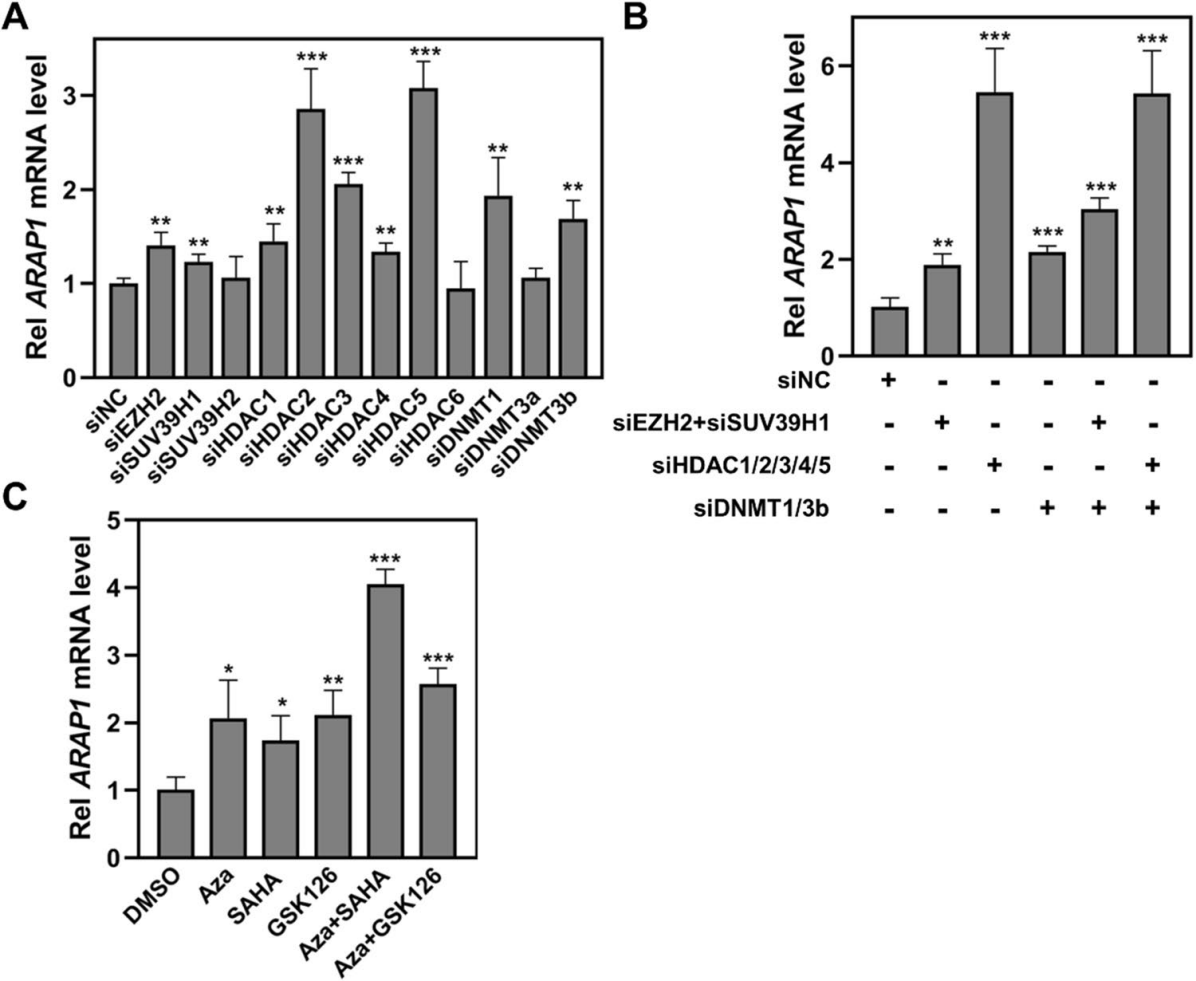
ARAP1 expression in LUAD is regulated by epigenetic modification. **A**, **B** A549 cells were transfected with indicated siRNA for 72 h, and the cells were collected and used for qRT-PCR to detect the expression of *ARAP1* mRNA. **C** A549 cells were treated with Aza (5 μM for 72 h), SAHA (2.5 μM for 24 h), GSK126 (5 μM for 72 h) alone or in combination. The cells were then collected and used for qRT-PCR to examine the expression of *ARAP1* mRNA. The value in each group was displayed as fold change relative to siNC or DMSO (mean ± SD, n = 3). *P < 0.05, **P < 0.01 and ***P < 0.001 compared to siNC by Student *t* test

Epigenetics plays an important role in controlling gene expression and has been demonstrated to participate in carcinogenesis [35]. To explore the mechanism of ARAP1 reduction in LUAD, we screened several epigenetic enzymes with suppressed function in genes expression and found that, except for other tested enzymes, knockdown of EZH2, SUV39H1, HDAC1, HDAC2, HDAC3, HDAC4, HDAC5, DNMT1 or DNMT3b could significantly induce an increase of *ARAP1* mRNA (Fig. 2A). We further demonstrated that simultaneously knockdown of above epigenetic enzymes can synergistically enhance *ARAP1* expression (Fig. 2B). Consistently, DNMT inhibitor (Aza), HDAC inhibitor (SAHA) or EZH2 inhibitor (GSK126) treatment could cause an increase of *ARAP1* mRNA, while combined treatment with these epigenetic inhibitors could synergistically upregulate *ARAP1* mRNA in A549 cells (Fig. 2C). Collectively, these results suggest that epigenetics modifications might be involved in ARAP1 expression controlling in LUAD cells.

### ARAP1 inhibits metastasis of LUAD in vitro and in vivo

**Fig. 3:**
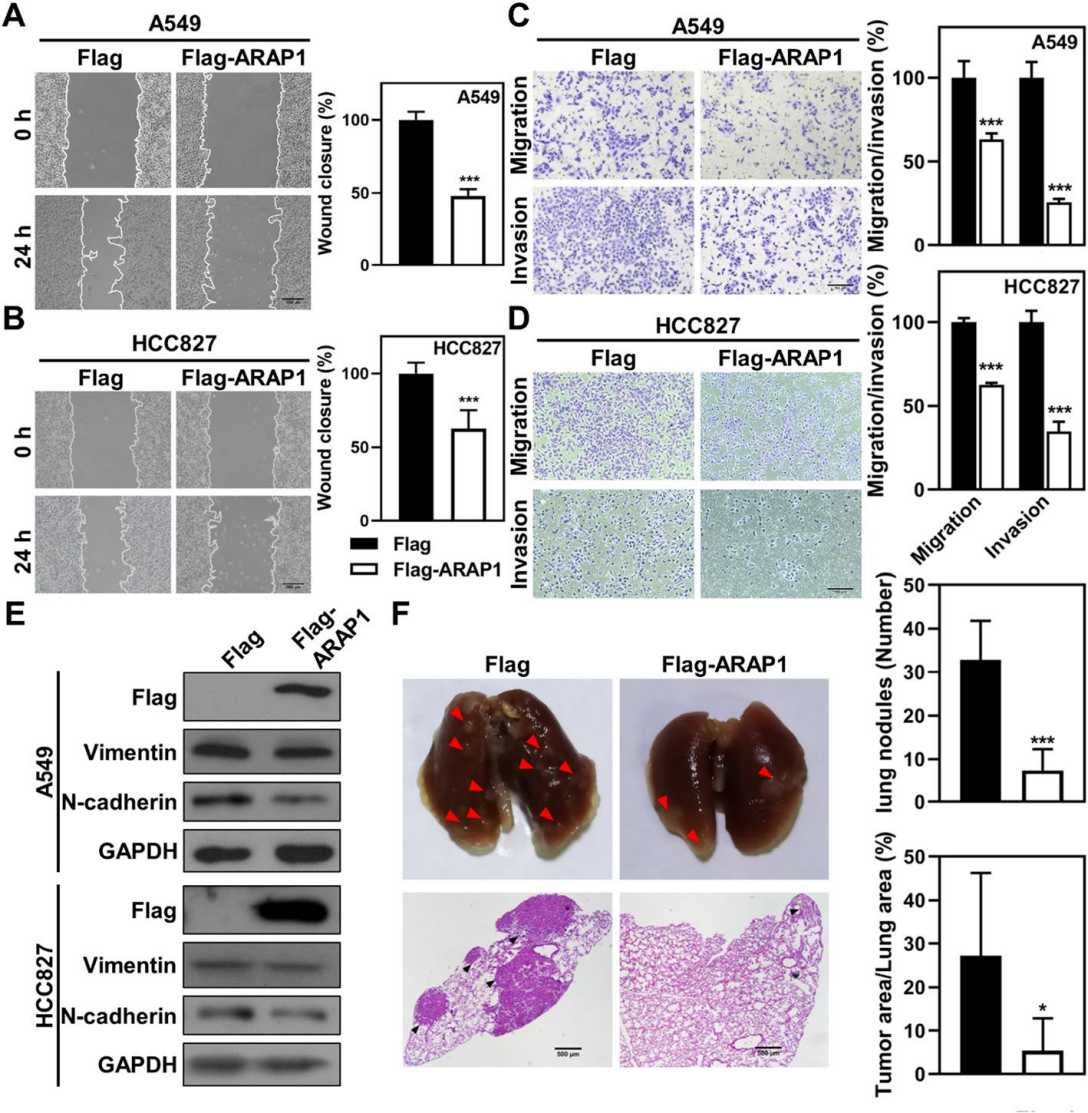
ARAP1 inhibits metastasis and EMT of LUAD cancer cells. Wound healing (**A**, **B**) and transwell (**C**, **D**) assays detected the effects of ARAP1 OE on migration and invasion of A549 cells (mean ± SD, n = 3). Scale bar: 200 μm. **E** Western blot analyzed EMT markers expression in LUAD cells after ARAP1 OE. **F** The effect of ARAP1 OE on metastasis of A549 cells in vivo. Red arrows indicate metastatic nodules on the lung surface and the tumor number is counted. The relative area of tumor to lung of each H&E image was calculated. Scale bar: 500 μm (n = 6). *P < 0.05 and ***P < 0.001 compared to control by Student *t* test

To investigate the effects of ARAP1 in LUAD, we constructed ARAP1 over-expressed (ARAP1 OE) LUAD cancer cell lines using a lentivirus system. As shown in Fig. S2A, B, lentivirus-mediated ARAP1 transduction significantly enhanced ARAP1 expression in A549 and HCC827 cells. CCK8 and colony formation assays revealed that ARAP1 OE had no significant effects on cell proliferation (Fig. S2C-H). We further assessed the effects of ARAP1 on cell migratory and invasive abilities. Wound-healing assays results showed that ARAP1 OE weakened the migratory ability of A549 and HCC827 cells (Fig. 3A, B). Transwell assays demonstrated that the number of migratory and invasive cells was significantly diminished when ARAP1 OE (Fig. 3C, D). Meanwhile, western blot and immunostaining results further revealed that ARAP1 OE caused a significantly decrease of mesenchymal markers N-cadherin and Vimentin in LAUD cells, and accompanied with a strikingly increased of epithelial marker E-cadherin (Fig. 3E and S3). Collectively, these results indicate that ARAP1 can impede EMT and metastasis in LUAD cells.

In vivo metastatic assay was also designed to examine the role of ARAP1 in metastasis. A metastatic model was established by tail vein injection of A549-ARAP1 OE cells and controls. The results showed that ARAP1 OE significantly decreased the number of pulmonary metastatic nodules in mice (Fig. 3F). Meanwhile, H&E-staining showed that ARAP1 OE induced a dramatically decreased size of metastatic nodules in the lung (Fig. 3F). In summary, these results demonstrate that ARAP1 could inhibit LUAD cancer cells metastasis.

### ARAP1 inhibits stress fibers formation in LUAD cancer cells

**Fig. 4:**
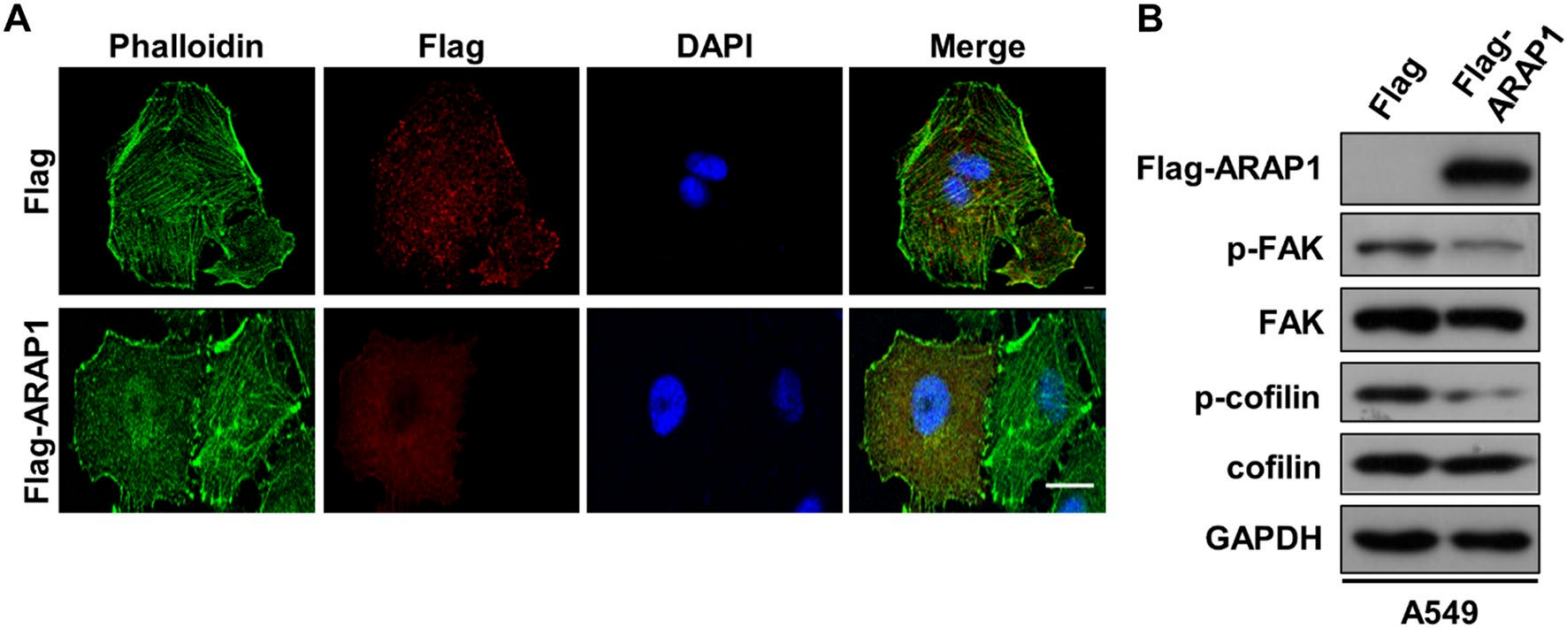
ARAP1 inhibits stress fibers formation. **A** Immunostaining analyzed stress fibers after ARAP1 OE. F-actin was labeled with phalloidin-FITC (green), Flag and Flag-ARAP1 transfected cells were labeled with anti-Flag antibody (red), and DNA was labeled with DAPI (blue). Scale bar: 10 μm. **B** Western blot examined the effects of ARAP1 OE on the expression of actin rearrangement related proteins and their phosphorylation including FAK and cofilin

Tumor cells undergoing EMT require rearrangements of actin cytoskeleton as well as formation of cell adhesion to satisfy the demand for cell retraction and motility [10]. Stress fiber composed of microfilament plays a critical role in regulating cells motility, migration and invasion [36]. We next sought to investigate whether ARAP1 regulates stress fibers formation in LUAD cancer cells. As shown in Fig. 4A, intracellular microfilament was stained with phalloidin-FITC, and large amount of robust stress fibers can be observed in control and vector transfected cells, while only scarce stress fibers were seen in ARAP1 OE cells. Since FAK (Focal adhesion kinase) regulates the organization of stress fibers via phosphorylating and inhibiting a severing protein, cofilin [37], we then detected the level of p-FAK and p-cofilin after ARAP1 OE. Western blot results showed that the level of p-FAK and p-cofilin was significantly decreased in ARAP1 OE cells (Fig. 4B and S4A). Therefore, we demonstrate that ARAP1 negatively regulates stress fibers formation, which would contribute to the motility and migration of LUAD cancer cells.

### ARAP1 regulates fibers formation via its RhoGAP activity

**Fig. 5:**
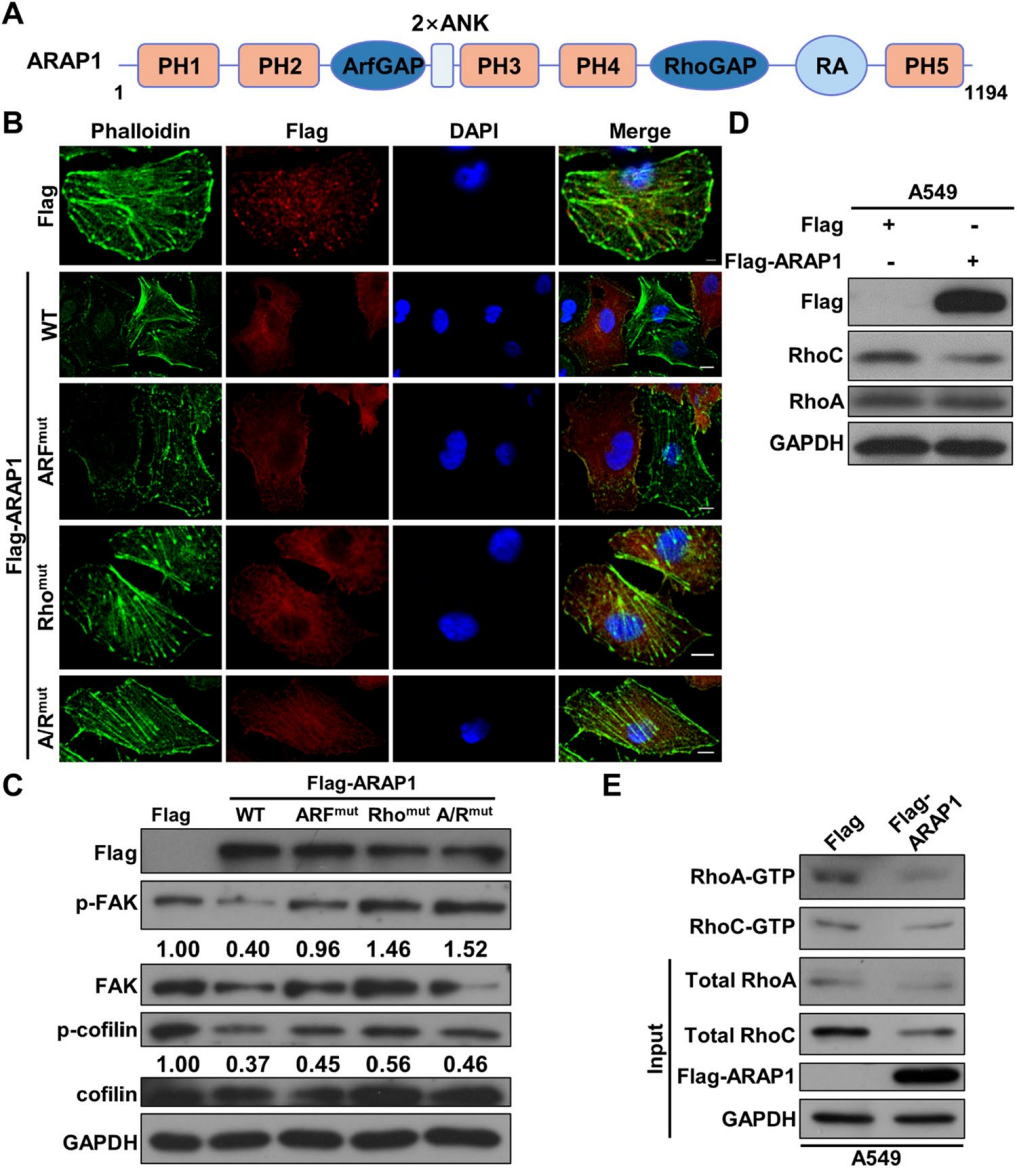
ARAP1 regulates stress fibers formation depending on its RhoGAP activity. **A** The schematic diagram of ARAP1 protein. **B** Immunostaining analyzed the effects of ARAP1 and its different mutants OE on stress fibers in A549 cells. DNA was labeled with DAPI. Scale bar: 10 μm. **C** Western blot examined the effects of ARAP1 and indicated mutants OE on the expression of actin rearrangement related proteins. The level of p-FAK and p-cofilin were quantified using Scion Image software and normalized to the density of the GAPDH bands. **D** Western blot detected the effects of ARAP1 OE on expression of Rho GTPases. **E** IP and western blot detected the effects of ARAP1 OE on Rho activity

ARAP1 is a GAP that has been reported to regulate Arf1/Arf5 activities via ArfGAP domain and Rho activities via RhoGAP domain (Fig. 5A) [21]. These small GTPases have been reported to control cell skeleton remodeling and cell metastasis [38]. Therefore, we wanted to determine which domain is responsible for ARAP1-induced inhibition of stress fibers formation. We constructed plasmids coding different GAP mutants and examined the effects of these mutants on the stress fibers. As shown in Fig. 5B, similar to ARAP1^WT^, overexpression of ARAP1 ARF^mut^ (ARAP1^R338K^ with ArfGAP mutation), but not ARAP1 Rho^mut^ (ARAP1^R753K^ with RhoGAP mutation) or ARAP1 A/R^mut^ (with both ArfGAP and RhoGAP mutation), dramatically inhibited stress fibers formation. Western blot results further demonstrated that, unlike WT and ARF^mut^, overexpression of Rho^mut^ or A/R^mut^ could not inhibit p-FAK and p-cofilin level (Fig. 5C). Therefore, we speculated that the inhibitory ability of ARAP1 to stress fibers formation attributes to its RhoGAP domain.

We next determined the effects of ARAP1 on Rho GTPases. Western blot results showed that ARAP1 OE suppressed the expression of RhoC but not RhoA in A549 cells, while inhibited the protein expression of RhoA but not RhoC in HCC827 cells (Fig. 5D and S4A). qRT-PCR results showed that ARAP1 OE did not cause similar effects on *RhoA/B/C* mRNA expression in different LUAD cancer cells (Fig. S4B, C). Therefore, we further wanted to examine whether ARAP1 affects Rho GTPases activity. Indeed, we found that the amount of activated RhoA-GTP and RhoC-GTP was reduced in ARAP1 OE cells (Fig. 5E). Altogether, these results imply that ARAP1 might affect microfilament remodeling via its RhoGAP activity.

### ARAP1-mediated metastatic inhibition mainly attributes to its RhoGAP domain

**Fig. 6:**
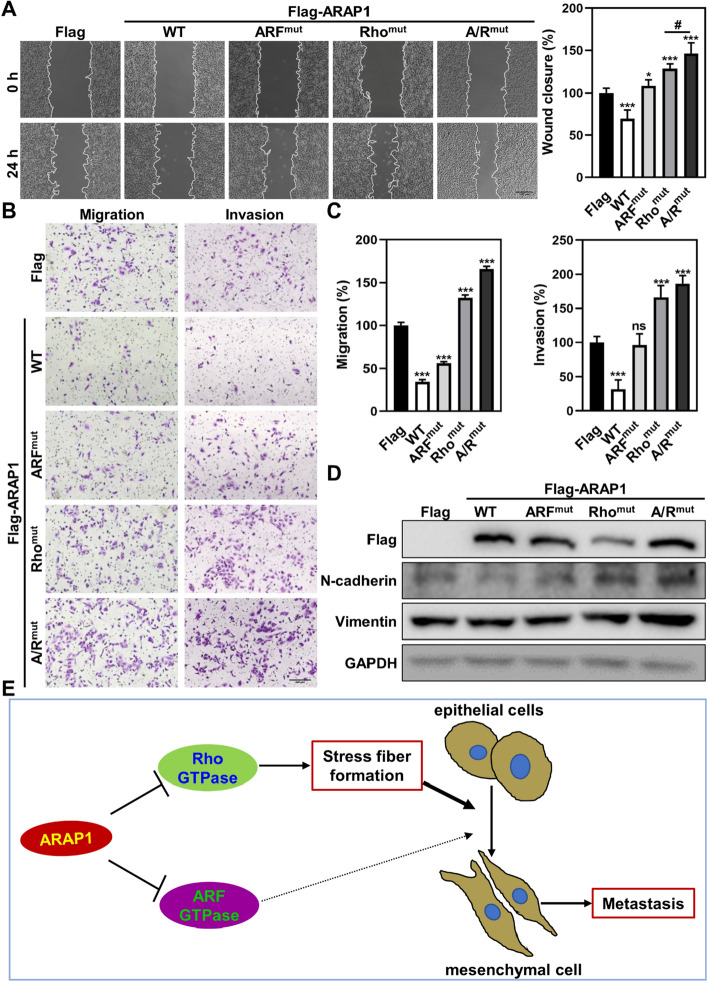
ARAP1-mediated inhibition of LUAD cancer cell metastasis is mainly dependent on its RhoGAP activity. Wound healing (**A**) and transwell (**B**, **C**) assays detected the effects of ARAP1 and indicated mutants OE on migration and invasion of A549 cells (mean ± SD, n = 3). Scale bar: 200 μm. **D** Western blot examined EMT markers expression after ARAP1 and indicated mutants OE. **E** The model of ARAP1 regulates metastasis in LUAD. ns: no significant; #*P* < 0.05; *P < 0.05 and ***P < 0.001 compared to control by Student *t* test

We further examined whether the GAP activity of ARAP1 contributes to its migratory and invasive inhibition in LUAD cancer cells. As shown in Fig. 6A-D, unlike ARAP1^WT^, the inhibitory ability of ARAP1 in cell migration, invasion and EMT was significantly impaired when ArfGAP or RhoGAP domain was mutated. In particular, compared with weaker impairment of ARF^mut^, Rho^mut^ and A/R^mut^ led to a significant promotion in metastasis of LUAD cancer cells. These results indicate that the RhoGAP activity contributes to the metastatic inhibition of ARAP1 to a greater extent.

## Discussion

Small GTPases regulate a range of cellular functions via different downstream effectors. Plenty of regulators have been demonstrated to modulate the activity of small GTPase and their abnormal expression and/or dysfunction are closely related to many human diseases, such as cancer [12, 39]. Here, we found that the expression of a GAP protein, ARAP1, is frequently reduced in tumor tissues and its downregulation is positively correlated with a worse prognosis in LUAD patients. ARAP1 serves as a metastatic suppressor mainly by inhibiting Rho GTPase-mediated stress fibers formation (Fig. 6E).

It is well known that solid tumors are heterogeneous and consisted of different types of cells, including cancer cells as well as endovascular cells, immune cells and mesenchymal cells [40]. In addition, cancer cells in a single tumor are also heterogeneous [41]. These features inevitably lead to the possibility that the expression of a biomarker is distinct among different patients. Here, we examined the expression of ARAP1 in a LUAD cohort, and found that ARAP1 is frequently downregulated in tumor tissues. To eliminate the effects of tumor heterogeneity, we also analyzed the expression of ARAP1 based on TCGA and CPTAC data, and the results further support that the expression of ARAP1 is reduced in LUAD tumor tissues. However, it is certainly worth examining the expression of ARAP1 and its relationship with the clinicopathological characteristics of LUAD patients in a large cohort.

It was known that epigenetics including DNA methylation and histone modification involved in regulating gene expression, which also plays a crucial role in tumorigenesis [42]. Some epigenetic regulators, such as DNMTs and HDACs, are upregulated in LUAD, which can inhibit the transcription of tumor suppressor genes, thus promoting LUAD progression [43, 44]. Meanwhile, these epigenetic regulators are often recruited onto a same promoter and synergistically modulate the transcription of these genes. For example, proto-oncoprotein FBI-1 recruits Mi-2, NuRD-HDAC complex as well as DNMTs, inhibiting the expression of *CDKN1A* [45]. Here, we found that inhibition of DNA methylation and histone modification could reverse the expression of *ARAP1* in LUAD cells, suggesting that these epigenetic modifications may participate in LUAD tumorigenesis by suppressing *ARAP1* expression. However, the epigenetic regulatory mechanism causing the downregulation of *ARAP1* in LUAD needs to further investigate.

Tumor metastasis, the leading reason for tumor-related mortality, remains a primary clinical problem in cancer diagnosis and treatment. To meet the requirement of motility and military, cytoskeleton particularly microfilaments are reorganized. It has been wildly proven that several specific microfilament-based structures including stress fibers, invadopodia, lamellipodia, filopodia and focal adhesion are involved in EMT and cell metastasis [46, 47]. Among them, stress fibers are dynamic structures shifted from cortical actin fibers that play important roles in cell motility and contractility. Our results found that ARAP1 significantly inhibited the stress fibers formation and metastasis in LUAD cancer cells, which further support the critical role of stress fiber in cell metastasis.

Rho family of GTPases acts as core regulators in cell migration. It has been reported that activated Rho, including Rho, Rac and Cdc42, can regulate microfilaments rearrangement and metastasis in different manners. For example, RhoA is required for stress fibers formation. Activated RhoA binds to and activates its downstream effectors ROCK 1/2, a kind of serine/threonine kinases, which subsequently activates LIMK. LIMK phosphorylates cofilin and inhibits cofilin-mediated actin filaments disassembly, therefore, facilitating stress fibers formation and cell motility [10, 48]. Additionally, RhoA/ROCK pathway could phosphorylate and activate FAK, which also participates in inactivating cofilin by promoting cofilin phosphorylation [37, 49]. Rac1 and Cdc42 regulate metastasis by promoting the lamellipodia and filopodia assembly, respectively [50, 51]. Dozens of GTPases regulators have been found to regulate Rho GTPase-mediated metastasis and play important role in carcinogenesis [52–58]. In this study, overexpression of ARAP1, a GAP protein, can effectively inhibit RhoA activity, which in turn suppresses the phosphorylation of FAK and cofilin, and then inhibits stress fibers formation and metastasis.

ARAP1 is a GAP protein that has been reported to inhibit Rho GTPase via RhoGAP domain and Arf1/5 GTPases activity via ArfGAP domain. Several studies have demonstrated that ARAP1 plays a critical role in regulating membrane trafficking and reorganization of actin cytoskeleton [20, 22, 23]. However, the functions of ARAP1 in different systems are controversial. For example, Hye-Young et al. found that ARAP1 knockdown accelerated the degradation of EGFR, while Tiziana et al. showed that ARAP1 silencing led to an accumulation of EGFR in a sorting/late endosomal compartment that is accompanied by prolonged EGFR signaling [22, 23]. Qin et al. found that overexpression of ARAP1 ArfGAP mutant, but not wild-type, could inhibit cell migration induced by shear stress treatment in MDA-MB-231 cells [59]. However, we demonstrated that ARAP1 could effectively inhibit stress fibers formation and metastasis in LUAD cells primarily by inhibiting RhoA and RhoC activities depending on the RhoGAP domain. It also cannot be ignored that Arf GAP activity of ARAP1 plays a certain role in regulating metastasis in LUAD cells, because we found that the ARF^mut^ impaired the capacity of ARAP in metastatic inhibition, although it did not affect the stress fibers formation.

In summary, we found that ARAP1 is frequently reduced in lung LUAD tumor tissues and cells, and displays metastatic suppression in LUAD cancer cells via inhibiting Rho GTPase-mediated microfilament remodeling.

### Supplementary Information

Additional file 1 (TIF 560 KB)

Additional file 2 (TIF 925 KB)

Additional file 3 (TIF 1039 KB)

Additional file 4 (TIF 408 KB)

Additional file 5 (DOCX 17 KB)

Additional file6 (DOCX 18 KB)

## Data Availability

The data and materials of the current study are available from the corresponding author upon reasonable request.

## Abbreviations

ARAP1: ArfGAP with RhoGAP domain, ankyrin repeat and PH domain 1
Arf: ADP ribosylation factor
CCK8: Cell counting kit 8
DNMT: DNA methyltransferase
EMT: Epithelial–mesenchymal transition
FAK: Focal adhesion kinase
FBI-1: Factor that binds to the inducer of short transcripts of human immunodeficiency virus-1
GAP: GTPase activating protein
GDI: Guanine nucleotide dissociation inhibitor
GEF: Guanine nucleotide exchange factor
HDAC: Histone deacetylase
LUAD: Lung adenocarcinoma
Mi-2: Mi-2 autoantigen
MLC: Myosin light chain
MYPT1: Myosin Phosphatase-Targeting Subunit 1
NSCLC: Non-small cell lung cancer
OS: Overall survival
NuRD: Nucleosome remodeling and histone deacetylase
PFS: Progression-free-survival
Rho: Ras homology
ROCK: Rho Kinase
SCLC: Small-cell lung cancer
