# Supplementary figures and images for: ARAP1 negatively regulates stress fibers formation and metastasis in lung adenocarcinoma via controlling Rho signaling
Source: Discov Oncol. 2023 Nov 27;14:214. doi: 10.1007/s12672-023-00832-x (PMC10678915; doi:10.1007/s12672-023-00832-x)

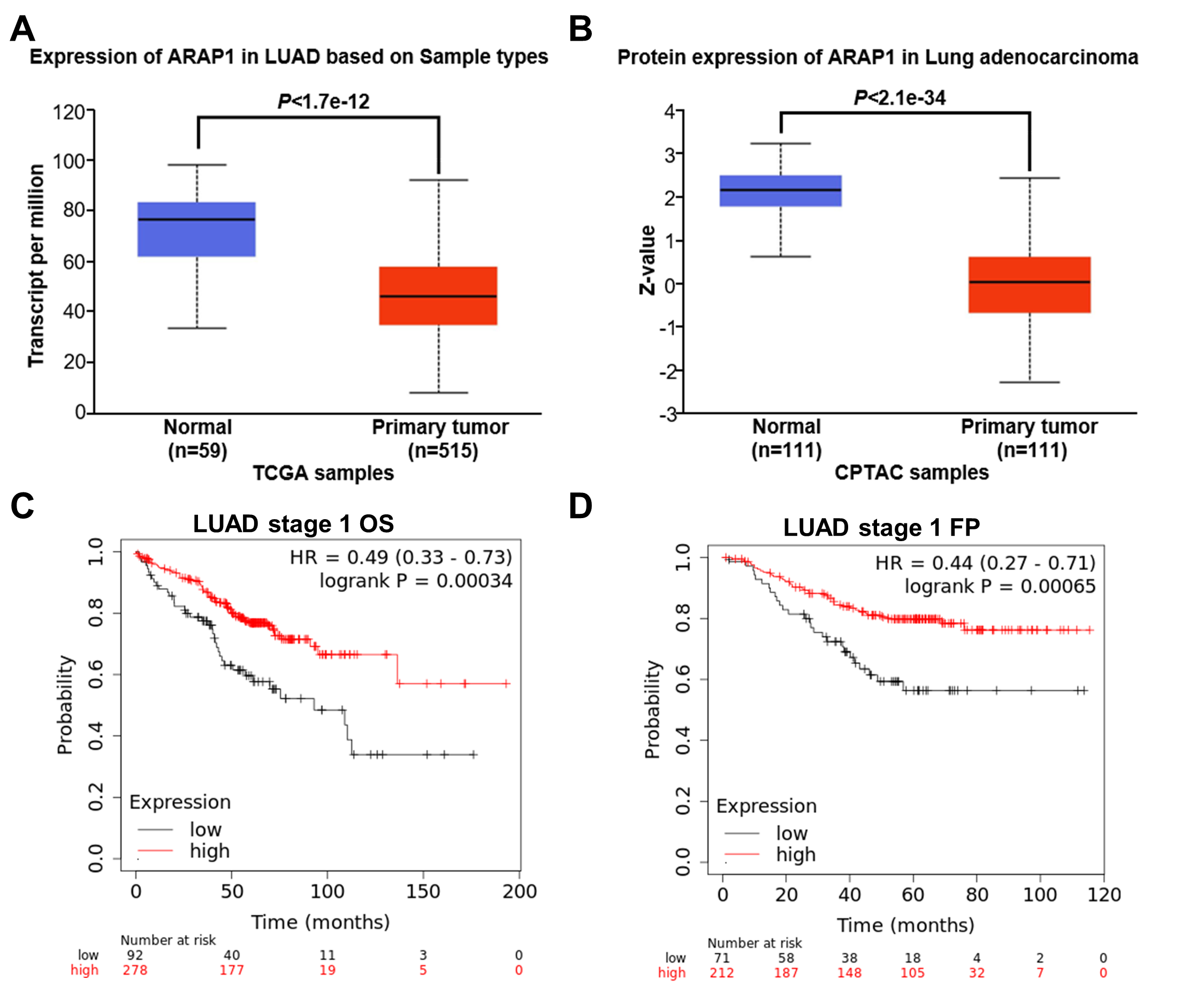

Supplement: Supplementary file 1 — Additional file 1 (TIF 560 KB) [file 12672_2023_832_MOESM1_ESM.tif]

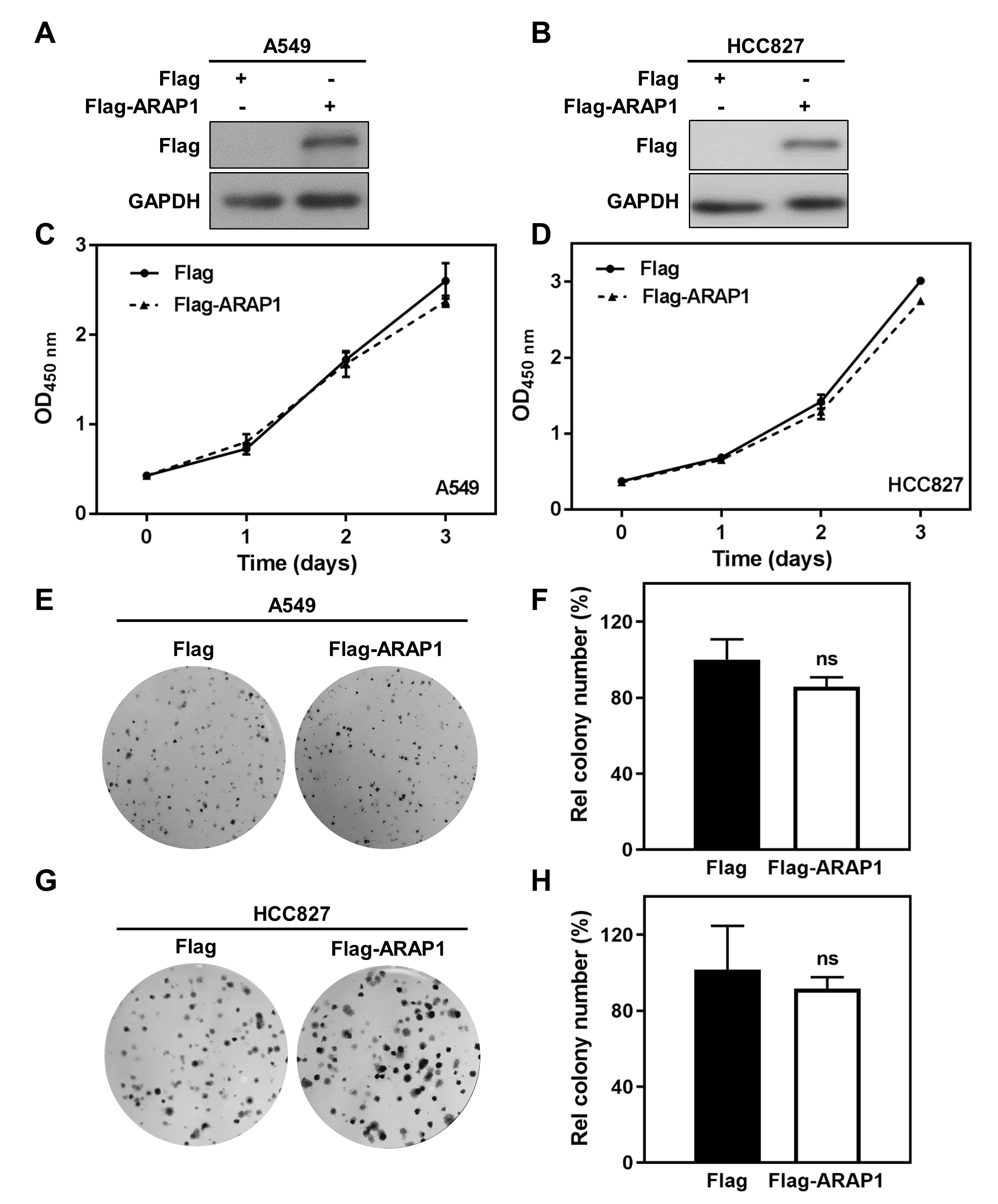

Supplement: Supplementary file 2 — Additional file 2 (TIF 925 KB) [file 12672_2023_832_MOESM2_ESM.tif]

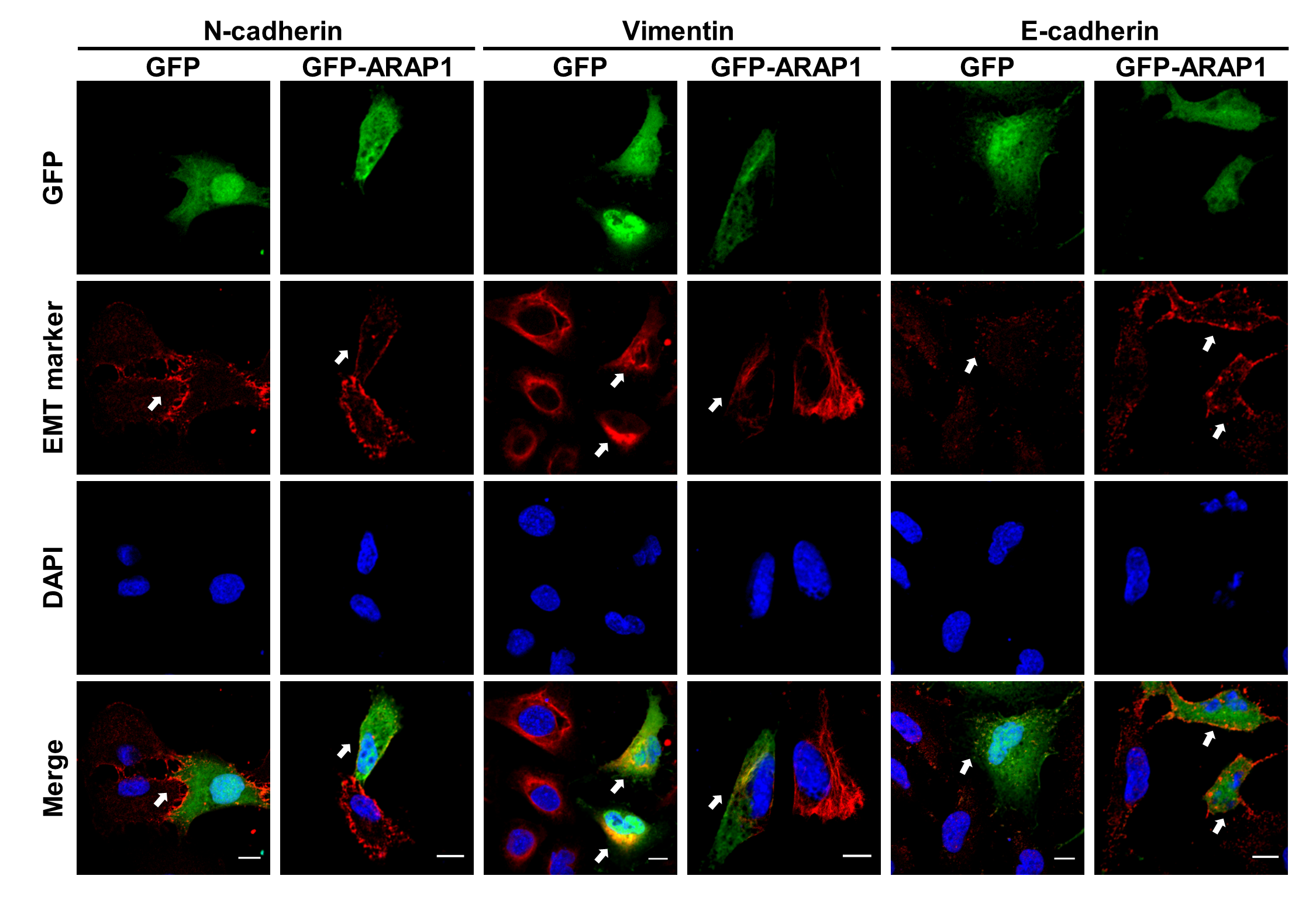

Supplement: Supplementary file 3 — Additional file 3 (TIF 1039 KB) [file 12672_2023_832_MOESM3_ESM.tif]

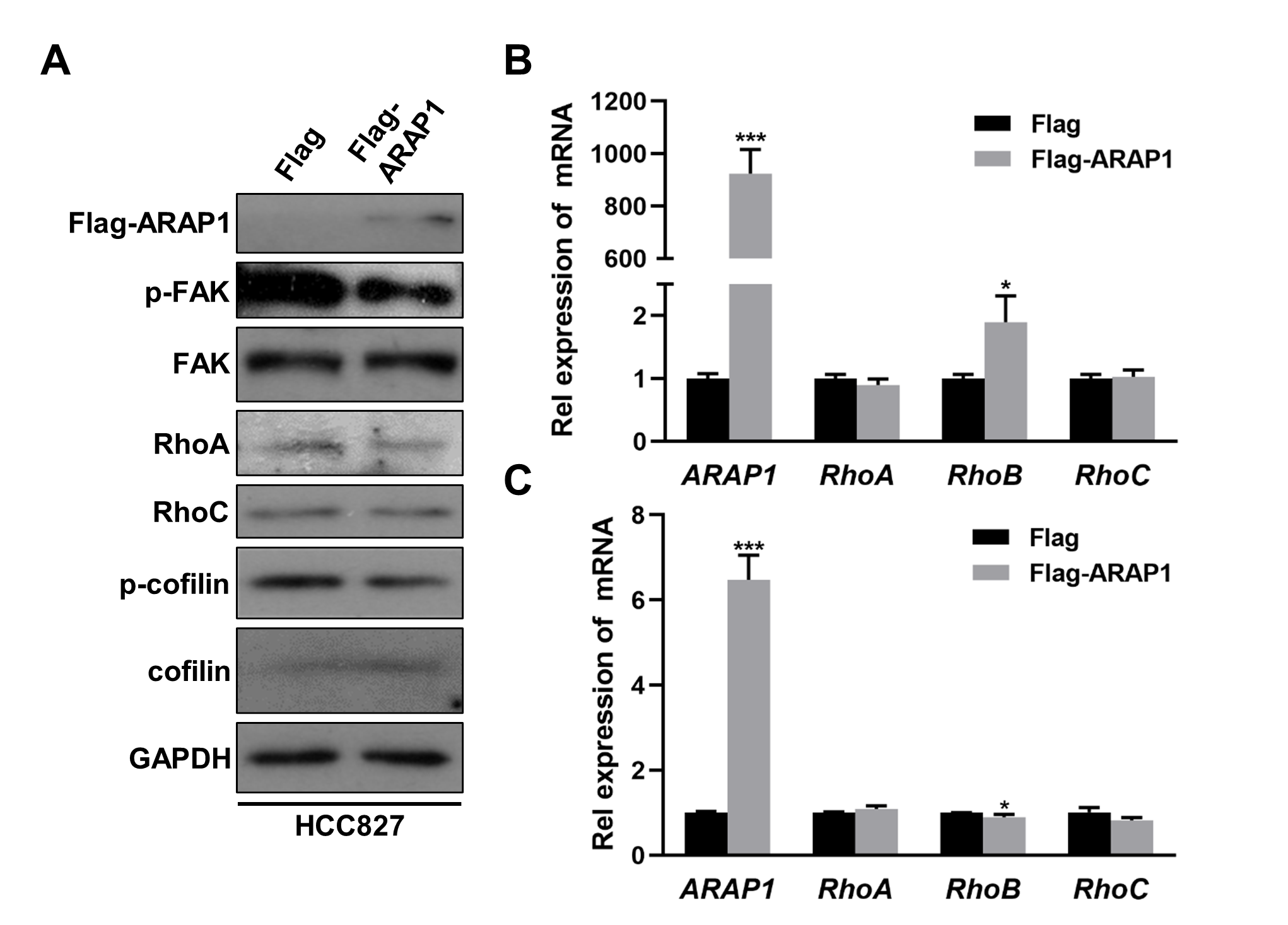

Supplement: Supplementary file 4 — Additional file 4 (TIF 408 KB) [file 12672_2023_832_MOESM4_ESM.tif]
