# Supplementary material for: ARAP1 negatively regulates stress fibers formation and metastasis in lung adenocarcinoma via controlling Rho signaling
Source: Discov Oncol. 2023 Nov 27;14:214. doi: 10.1007/s12672-023-00832-x (PMC10678915; doi:10.1007/s12672-023-00832-x)
Supplement: Supplementary file 5 — Additional file 5 (DOCX 17 KB) [file 12672_2023_832_MOESM5_ESM.docx]

Table S1. The information of antibodies.

| Antibodies | Source | Catalogue | Dilution |
| --- | --- | --- | --- |
| anti-N-cadherin | Proteintech | 22018-1-AP | 1:2000 (1:100 for immunofluorescence) |
| anti-E-cadherin | Proteintech | 20874-1-AP | 1:5000 (1:100 for immunofluorescence) |
| anti-Vimentin | Proteintech | 10366-1-AP | 1:2000 (1:100 for immunofluorescence) |
| anti-cofilin | Proteintech | 66057-1-Ig | 1:10000 |
| anti-FAK | Proteintech | 66258-1-Ig | 1:1000 |
| anti-GAPDH | Proteintech | 60004-I-Ig | 1:10000 |
| anti-RhoA | Cell Signaling Technology | 2117S | 1:1000 |
| anti-RhoC | Cell Signaling Technology | 3430S | 1:1000 |
| anti-phospho-cofilin | Cell Signaling Technology | 3313S | 1:1000 |
| anti-phospho-FAK | Cell Signaling Technology | 3283S | 1:1000 |
| anti-Flag | Sigma-Aldrich | F1804 | 1:1000 |
| anti-ARAP1 | Invitrogen | PA5-67101 | 1:1000 |
| Goat anti-Rabbit HRP | Life Technologies Inc. | 31460 | 1:10000 |
| Goat anti-Mouse HRP | Life Technologies Inc. | 31430 | 1:10000 |
| Goat Anti-Rabbit IgG Alexa Fluor 546 | Invitrogen | A-11035 | 1:300 |
| Goat Anti-mouse IgG Alexa Fluor 546 | Invitrogen | A-11030 | 1:300 |
