# Supplementary material for: ARAP1 negatively regulates stress fibers formation and metastasis in lung adenocarcinoma via controlling Rho signaling
Source: Discov Oncol. 2023 Nov 27;14:214. doi: 10.1007/s12672-023-00832-x (PMC10678915; doi:10.1007/s12672-023-00832-x)
Supplement: Supplementary file 6 — Additional file6 (DOCX 18 KB) [file 12672_2023_832_MOESM6_ESM.docx]

Table S2. Expression of *ARAP1* in lung adenocarcinoma tissues (n = 38)

| Variables | *ARAP1* expression | | χ^2^ | *P* value |
| --- | --- | --- | --- | --- |
|  | Low | High |  |  |
| Gender |  |  | 10.795 | 0.001 |
| Female | 3 | 13 |  |  |
| Male | 16 | 6 |  |  |
| Age (years) |  |  | 0.585 | 0.444 |
| <62 | 9 | 7 |  |  |
| ≥62 | 10 | 12 |  |  |
| Localization, n |  |  | 1.067 | 0.587 |
| Left lung | 11 | 11 |  |  |
| Right lung | 7 | 8 |  |  |
| Both lungs | 0 | 1 |  |  |
| Lymph node metastasis, n |  |  | 0.110 | 0.500 |
| Yes | 7 | 8 |  |  |
| No | 12 | 11 |  |  |
| TNM stage |  |  | 5.183 | 0.075 |
| I | 8 | 4 |  |  |
| II | 2 | 8 |  |  |
| III | 7 | 9 |  |  |
